# Adrenocortical carcinoma initially presenting with hypokalemia and hypertension mimicking hyperaldosteronism: a case report

**DOI:** 10.1186/1756-0500-6-405

**Published:** 2013-10-08

**Authors:** Chun-Jui Huang, Ti-Hao Wang, Yuan-Hao Lo, Kuan-Ting Hou, Justin Ging-Shing Won, Tjin-Shing Jap, Chin-Sung Kuo

**Affiliations:** 1 Division of Endocrinology and Metabolism, Department of Medicine, Taipei Veterans General Hospital, No. 201, Sec. 2, Shih-Pai Rd, Taipei 112, Taiwan; 2 Cancer Center, Taipei Veterans General Hospital, No. 201, Sec. 2, Shih-Pai Rd, Taipei 112, Taiwan; 3 Department of Medicine, National Yang-Ming University, No. 155, Sec. 2, Linong Street, Taipei 112, Taiwan; 4 Institute of Clinical Medicine, National Yang-Ming University, No. 155, Sec. 2, Linong Street, Taipei 112, Taiwan

**Keywords:** Adrenocortical carcinoma, Cushing’s syndrome, Hyperaldosteronism

## Abstract

**Background:**

Adrenocortical carcinoma is a rare malignancy and rare cause of Cushing’s syndrome.

**Case presentation:**

A 65-year-old seemingly well male patient was referred to our clinic under the suspicion of hyperaldosteronism due to hypertension combined with hypokalemia. However, his serum aldosterone and plasma renin activity were within normal limits. Instead, Cushing’s syndrome was diagnosed by elevated urine free cortisol and a non-suppressible dexamethasone test. Abdominal computed tomography showed a 7.8 × 4.8 cm mass lesion at the right adrenal gland with liver invasion. Etomidate infusion was performed to reduce his cortisol level before the patient received a right adrenalectomy and liver wedge resection. The pathology report showed adrenocortical carcinoma with liver and lymph node metastasis. According to the European Network for the Study of Adrenal Tumors (ENSAT) staging system, the tumor was classified as T4N1M1, stage IV. Recurrent hypercortisolism was found shortly after surgery. The patient died of Fournier’s gangrene with septic shock on the 59^th^ day after diagnosis.

**Conclusions:**

We report a case of rapidly progressive stage IV adrenocortical carcinoma with initial presentations of hypokaelmia and hypertension, mimicking hyperaldosteronism.

## Background

Adrenocortical carcinoma (ACC) is a rare malignancy, with an incidence of 0.7-2.0/million population/year [1]. Approximately 50 to 60% of cases of ACC present with an excess of adrenal steroid hormone, with the most common form being Cushing’s syndrome with hypercortisolism. Less frequently, an excess of sex hormones, including androgen or estrogen, may cause virilization in women and feminization in men [1]. Hypertension and profound hypokalemia may be due to mineralocorticoid excess. However, true hyperaldosteronism is rare [2].

The clinical presentations of ACC are heterogeneous and the prognosis is generally poor. Although ACCs are often hormonally active with glucocorticoid over-secretion, it is a rare cause of Cushing’s syndrome [1]. Here, we report a case of ACC with initial presentations of hypokalemia and hypertension mimicking hyperaldosteronism.

## Case presentation

**Figure 1:**
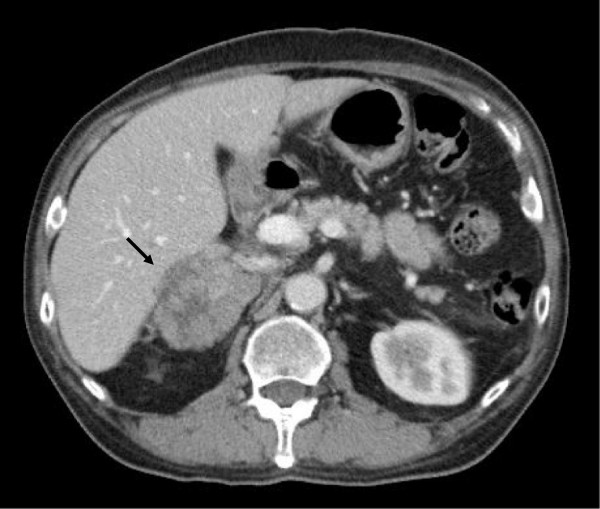
**Abdomen contrast computed tomography (CT) showed a 7.8 × 4.8 cm heterogenous lobulated enhancing mass lesion (arrow) at right adrenal gland with suspicious local invasion to the posterior segment of liver.**

**Figure 2:**
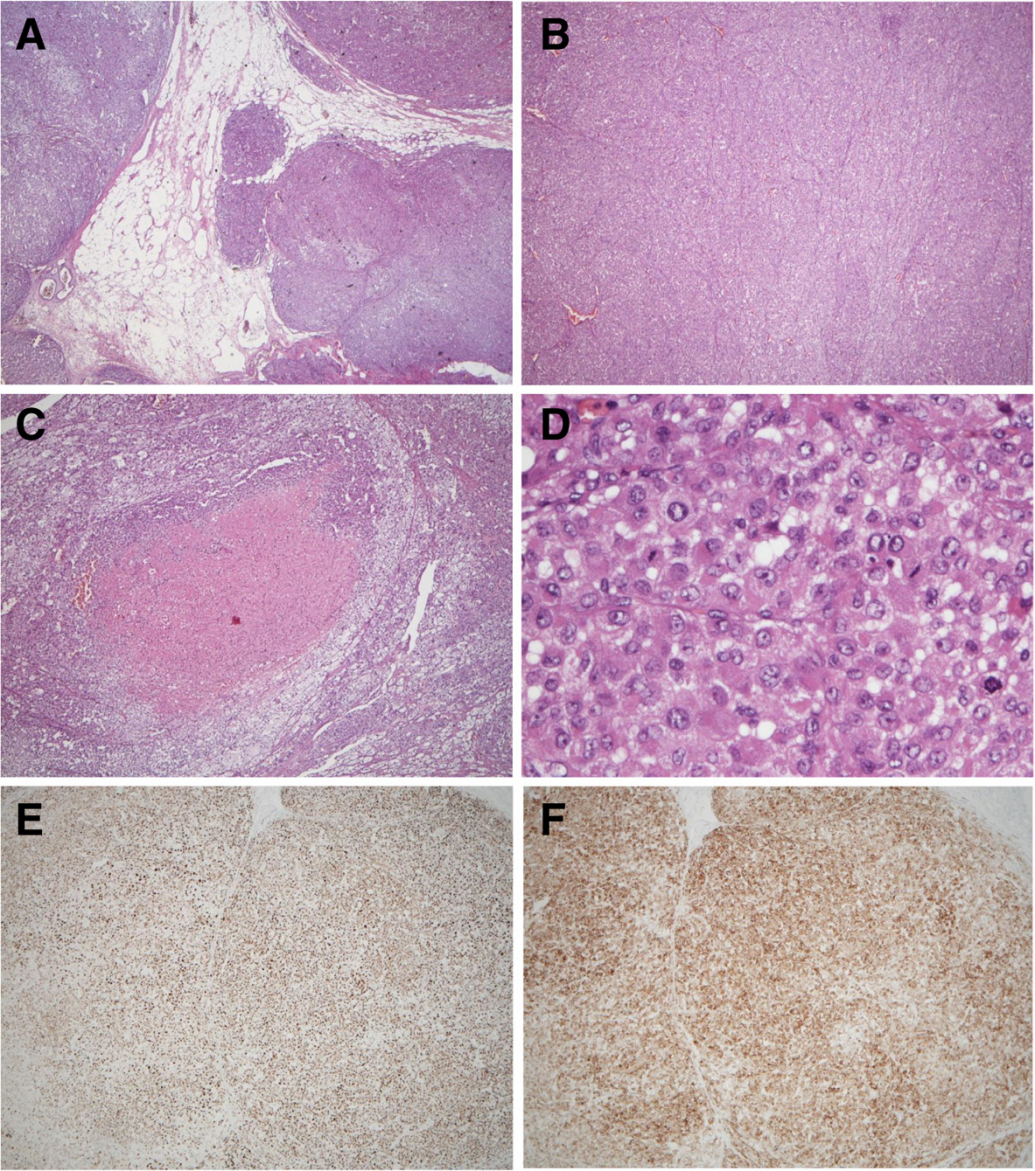
The pathological findings of the tumor showed (A) sinusoidal and capsular invasion with irregular border (hematoxylin and eosin, 40×), (B) sheet-like arrangement (40×), (C) central necrosis (100×), (D) high grade nucleus with frequent mitoses (400×), and positive immunohistochemical staining for both (E) melan A and (F) alpha-inhibin.

A 65-year-old seemingly well male was referred to our clinic due to hypertension and hypokalemia. He denied any discomfort in the past few months. However, vague symptoms of muscle weakness with cramping, polyuria, and nocturia were noted upon further interview. During his annual health check-up six months ago, he was diagnosed with hypertension and type 2 diabetes mellitus. On physical examination, blood pressure was 148/72 mmHg. There were no purple striae, central obesity, buffalo hump, proximal muscle weakness, or skin hyperpigmentation. His serum potassium level was 2.2 mmol/l. Hormonal studies demonstrated excess of serum cortisol with loss of diurnal pattern, elevated urine free cortisol and a non-suppressible dexamethasone test. The aldosterone level and plasma renin activity were in normal limits. The laboratory results of hormonal studies are summarized in Table 1. Abdominal computed tomography (CT) showed a 7.8 × 4.8 cm heterogenous lobulated enhancing mass lesion at the right adrenal gland with suspicious local invasion to the posterior segment of the liver (Figure 1). To reduce the cortisol level before operation, etomidate 2 mg/hr was infused and the serum cortisol level decreased from 59.95 μg/dl to 13.3 μg/dl in 48 hours. One week after diagnosis, he received surgical removal of his right adrenal tumor, retrocaval lymph nodes and wedge resection of the liver. Grossly, it was a bulky tumor, measuring 11.6 × 7.0 × 5.1 cm in size with irregular border and central necrosis. Microscopic examination revealed a sheet architectural pattern and focal necrosis. The neoplastic cells had acidophilic cytoplasm with pleomorphism, hyperchromasia, and high nuclear grade. There was sinusoidal and capsular invasion. The immunohistochemical stains revealed that the tumor cells were immunoreactive for melan A and alpha-inhibin diffusely and for calretinin focally. Based on the Weiss system, the tumor was classified as adrenocortical carcinoma. The liver and the retrocaval lymph nodes showed evidence of metastasis (Figure 2). According to the European Network for the Study of Adrenal Tumors (ENSAT) staging system, the tumor was classified as T4N1M1, stage IV. Shortly after the operation, the patient presented with severe sepsis with recurrent hypercortisolism. The patient and his family refused further therapy under critical status and the patient died as a result of Fournier’s gangrene with septic shock on the 59^th^ day after diagnosis.

**Table 1:** Results of hormone studies

**Hormone**	**Concentration**	**Reference**
Basal hormones		
08:00 AM Serum Cortisol (μg/dl)	40.75	5–25
22:00 PM Serum Cortisol (μg/dl)	37.28	<5
08:00 AM ACTH (pg/dl)	<5	<46
22:00 PM ACTH (pg/dl)	<5	<46
Urinary free cortisol (μg/day)	2584	20–80
Plasma renin activity (pg/ml)	14.81	3–33
Plasma aldosterone (pg/ml)	270	10–310
Urinary VMA (mg/day)	3.9	1.0–7.0
Serum DHEA-S (μmol/l)	2.47	2.49–13.9
High-dose dexamethasone test^a^		
Serum cortisol (μg/dl)	59.85	<46
Urinary free cortisol (μg/day)	4380	20–80

ACTH, adrenocorticotrophic hormone; VMA, vanillylmandelic acid; DHEA-S, dehydroepiandrosterone sulfate.

^a^Dexamethasone (2 mg) was taken orally every 6 h at 9 AM, 3 PM, 9 PM and 3 AM for a total of eight doses. Blood was drawn 4 h after the last dose and 24 h urine was collected for measurement of the urine free cortisol level from 9 AM on the second day to 9 AM on the third day of dexamethasone administration.

## Discussion

The patient was initially referred to our clinic under the suspicion of hyperaldosteronism due to hypokalemia and new onset hypertension. Nevertheless, his serum aldosterone and plasma renin activity were within normal limits. Instead, Cushing’s syndrome was confirmed by loss of diurnal cortisol and adrenocorticotrophic hormone (ACTH) patterns, markedly elevated urine free cortisol level and a non-suppressible dexamethasone test. Excessive cortisol overwhelms the capacity of 11β-hydroxysteroid dehydrogenase type 2 in the proximal tubule to inactivate cortisol to cortisone, thus allowing cortisol to saturate the mineralocorticoid receptor, causing severe hypokalemia in our patient [3].

The image features suggestive of ACC in our case included large tumor size (> 4 cm) with irregular margins, heterogeneous appearance, and local invasion of the liver [4]. Some authors suggested that magnetic resonance imaging (MRI) is superior in assessing the extent of liver metastasis and invasion into adjacent organs and vessels, especially thrombi in the inferior vena cava [5]. MRI also offers additional information on chemical shift imaging in the differentiation of benign or malignant lesions [6,7]. However, MRI as an initial imaging modality is less standardized probably due to its high expense. Recently, fluorodeoxyglucose-positron emission tomography (FDG-PET) has emerged as a useful adjuvant method in differentiating adrenal lesions. High 18 F-FDG uptake implies increased glucose metabolism and suggests malignancy [8]. Nevertheless, it is not yet a part of routine clinical practice.

Differentiating ACC from benign adenoma microscopically is a challenging issue even for experienced pathologists. The Weiss System has been the standard method for decades. Fulfilling more than three of the nine following histologic features is regarded as malignancy: (1) high nuclear grade 3 or 4; (2) mitotic rate greater than 5/50 high-power fields (HPF); (3) atypical mitoses; (4) tumors with 25% or less clear cells; (5) diffuse architecture more than 1/3; (6) microscopic necrosis; and (7) venous, (8) sinusoidal, and (9) capsular invasion [9]. It was not until recently that a modified Weiss System was proposed. The modified system aimed to eliminate the subjective items and suggested a score of ≥3 (2 × mitotic rate > 5/50HPF + 2 × clear cells < 25% cytoplasm + abnormal mitoses + necrosis + capsular invasion) as having malignant behavior [10]. Our patient scored 7 in the Weiss System and 5 in the modified Weiss System, both confirming the malignant nature of the disease.

Management for ACC requires a multidisciplinary approach. The principal considerations are surgical, which is the only curative option for ACC. For cortisol-secreting tumors with a urine free cortisol level more than 700–800 μg/day, short-term use of etomidate for rapid control of cortisol level before operation was performed in our Endocrine Unit to minimize surgical risk such as post-operation infection and poor wound healing. Etomidate as a steroidogenesis inhibitor, is an imidazole derivative that was initially used for rapid hypnosis induction [11]. Its use for rapid sequence intubation in septic patients was associated with increased mortality and adrenal insufficiency according to a recent meta-analysis [12]. Based on small series, case report studies and consensus guidelines, hypercortisolism may present as a medical emergency with severe metabolic disarrangement and the use of etomidate for rapid control of hypercortisolism is generally safe if closely monitored and titrated in the hands of experienced endocrinologists [13]. The recommended infusion rate for hypercortisolism was 0.04-0.05 mg/kg/hr, a dose much lower than when used to induce anesthesia. While it rarely causes hepatotoxicity, dose adjustment in the elderly and patients with renal insufficiency is needed [13].

The treatment of non-resectable or recurrent tumor relies on mitotane-based chemotherapy, albeit a low response rate [14]. Our patient presented with overt biochemical Cushing’s syndrome without the classical clinical features, which suggests a rapidly progressing tumor. Combination therapy with mitotane plus EDP (etoposide, doxorubicin and cisplatin) may be considered according to the results of the FIRM-ACT study [15]. The 5-year survival rate of stage IV ACC is less than 25% despite treatment. Poor prognostic factors including old age, high stage, and cortisol hypersecretion all existed in our patient, predicting a lesser chance of survival despite aggressive therapy [16].

## Conclusions

We report a case of rapidly progressive stage IV ACC with liver metastasis and cortisol excess in a seemingly well individual with hypertension and hypokalemia. Poor prognosis was anticipated in our patient with old age, high stage, and cortisol hypersecretion.

## Consent

Written informed consent was obtained from the patient’s next-of-kin for publication of this case report and any accompanying images. A copy of the written consent is available for review by the Editor-in-Chief of this journal.

## Abbreviations

ACC: Adrenal cortical carcinoma; CT: Computed tomography; ACTH: Adrenocorticotrophic hormone; MRI: Magnetic resonance imaging; FDG-PET: Fluorodeoxyglucose-positron emission tomography.

## Competing interests

The authors declare that they have no competing interests.

## Authors’ contributions

CJH and THW analyzed and interpreted the patient’s data and wrote the manuscript. CSK revised the manuscript. THW, YHL, KTH, GSW, TSJ, CSK managed the patient clinically. All authors read and approved the final manuscript.
